# *catena*-Poly[[dibromidomercury(II)]-μ-3,6-bis­(2-pyridyl­sulfan­yl)pyridazine-κ^2^ *N* ^3^:*N* ^6^]

**DOI:** 10.1107/S1600536811028066

**Published:** 2011-07-23

**Authors:** Xue-Hua Zhu, Xiao-Yuan Yang, Rui-Feng Song, Hai-Yan Li

**Affiliations:** a School of Chemistry and Bioengineering, Suzhou University of Science and Technology, Suzhou 215009, People’s Republic of China; b College of Chemistry, Chemical Engineering and Material Science, Suzhou University, Suzhou 215123, People’s Republic of China

## Abstract

In the title coordination polymer, [HgBr_2_(C_14_H_10_N_4_S_2_)]_*n*_, the Hg^II^ atom is four-coordinated in a distorted tetra­hedral geometry by the two N atoms of the pyridyl groups of different 3,6-bis­(2-pyridyl­sulfan­yl)pyridazine ligands and two Br atoms. The bridging function of the *cis* ligands leads to a helical chain structure along [100].

## Related literature

For metal coordination compounds with 3,6-bis­(2-pyridyl­thio) pyridazine, see: Chen *et al.* (1996 ▶); Mandal *et al.* (1987 ▶, 1988 ▶); Song *et al.* (2011 ▶); Woon *et al.* (1986 ▶).
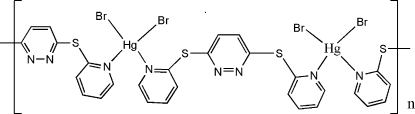


## Experimental

### 

#### Crystal data

[HgBr_2_(C_14_H_10_N_4_S_2_)]
*M* *_r_* = 658.78
Monoclinic, 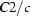

*a* = 16.393 (3) Å
*b* = 12.4954 (19) Å
*c* = 9.7648 (16) Å
β = 117.444 (3)°
*V* = 1775.1 (5) Å^3^
*Z* = 4
Mo *K*α radiation
μ = 13.41 mm^−1^
*T* = 223 K
0.55 × 0.30 × 0.26 mm

#### Data collection

Rigaku Saturn diffractometer
Absorption correction: multi-scan (*REQAB*; Jacobson, 1998 ▶) *T*_min_ = 0.013, *T*_max_ = 0.030
5341 measured reflections
2013 independent reflections
1688 reflections with *I* > 2σ(*I*)
*R*_int_ = 0.032

#### Refinement

*R*[*F* ^2^ > 2σ(*F* ^2^)] = 0.038
*wR*(*F* ^2^) = 0.080
*S* = 1.00
2013 reflections
107 parameters
H-atom parameters constrained
Δρ_max_ = 1.56 e Å^−3^
Δρ_min_ = −2.20 e Å^−3^

### 

Data collection: *CrystalClear* (Rigaku, 2001 ▶); cell refinement: *CrystalClear*; data reduction: *CrystalStructure* (Rigaku, 2001 ▶); program(s) used to solve structure: *SHELXS97* (Sheldrick, 2008 ▶); program(s) used to refine structure: *SHELXL97* (Sheldrick, 2008 ▶); molecular graphics: *SHELXTL* (Sheldrick, 2008 ▶); software used to prepare material for publication: *SHELXTL*.

## Supplementary Material

Crystal structure: contains datablock(s) I, global. DOI: 10.1107/S1600536811028066/hg5063sup1.cif

Structure factors: contains datablock(s) I. DOI: 10.1107/S1600536811028066/hg5063Isup2.hkl

Additional supplementary materials:  crystallographic information; 3D view; checkCIF report

## Figures and Tables

**Fig. 1.**
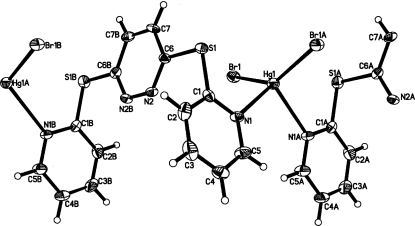
The the coordination environment of HgII center of complex(I), with atom labels and 30% probability displacement ellipsoids for non-H atoms.

**Fig. 2.**
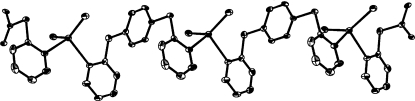
Perspective view of the 1-D helical chain structure of complex(I).

## supplementary crystallographic information

### Comment

In recent years, metal complexes of N-containing heterocyclic flexible thioethers ligands especially attracted considerable interest. The ligand 3,6-bis(2-pyridylthio) pyridazine (PTP) is interesting bridging ligand and is able to act as bridges between metal centers to form dinuclear (Chen *et al.*, 1996; Mandal *et al.*, 1987, 1988; Woon *et al.*, 1986) and coordination polymer (Song *et al.* 2011). Herein, we report the crystal structure of Hg^II^ complex (I).

The title complex (I) is one-dimensional chain coordination polymer. Each Hg^II^ atom is in a distorted tetrahedral geometry with two N atoms from two different PTP ligands and two bromide (Fig. 1). Its structure is isomorphous with HgI_2_ complex (Song *et al.*, 2011). Complex (I) has a compressed N—Hg—N angle of 98.8 (2)° and an expanded Br—Hg—Br angle of 140.84 (4)°, whereas for iodide structure the compressed N—Hg—N angle and expanded I—Hg—I angle are 96.6 (2)° and 144.01 (2)° (Song *et al.*, 2011), respectively. As HgI_2_ complex (Song *et al.*, 2011), HgBr_2_ units of the complex (I) are connected to each other by *cis*-PTP ligands through the pyridyl nitrogen atoms into a one-dimensional chain along [100] (Fig. 2). The ligand adopts a pronounced *syn* twist, creating an angle of 15.3 (1)° between the pyridine planes and angles of 83.2 (1)° between the pyridine planes and the pyridazine plane. The two pyridyl groups in PTP are not coplanar, and the bending of the ligand and its coordination at the Hg(II) center result in one-dimensional chains that adopt a helical twist. The isomorphous structure results from tetrahedral coordination geometry Hg^II^ ions.

### Experimental

The ligand 3,6-bis(2-pyridylthio) pyridazine (PTP) was prepared according to the general procedure reported by Woon *et al.* (1986). For preparation of the title compound, a solution of HgBr_2_ (18.5 mg, 0.05 mmol) in acetone (2 ml) was slowly added to a solution of PTP (15 mg, 0.05 mmol) in CH_3_OH (2 ml). The mixture was stirred for 0.5 h at room temperature, and then filtered and kept in the refrigerator (-18 C°). After 48 h, yellow prismatic single-crystals (I) of suitable for X-ray analysis was obtained in 67.2% yield. IR (cm^-1^): 3039.81.w, 2368.58w, 1581.62*m*, 1450.47*m*, 1427.32*m*, 1396.46 s, 833.25w, 771.52 s, 632.65w, 578.64w. Anal. Found: C, 25.53; H, 1.51; N, 8.46. Calcd. For C_14_H_10_Br_2_HgN_4_S_2_: C, 25.50; H, 1.32; N, 8.50.

### Refinement

H atoms were included in calculated positions refined as part of a riding with C—H distances of 0.94Å (aromatic H), and with *U*_iso_= 1.2*U*_eq_(C).

### Crystal data

**Table:**

[HgBr_2_(C_14_H_10_N_4_S_2_)]	*F*(000) = 1216
*M**_r_* = 658.78	*D*_x_ = 2.465 Mg m^−^^3^
Monoclinic, *C*2/*c*	Mo *K*α radiation, λ = 0.71075 Å
Hall symbol: -C 2yc	Cell parameters from 2664 reflections
*a* = 16.393 (3) Å	θ = 3.6–27.5°
*b* = 12.4954 (19) Å	µ = 13.41 mm^−^^1^
*c* = 9.7648 (16) Å	*T* = 223 K
β = 117.444 (3)°	Prism, yellow
*V* = 1775.1 (5) Å^3^	0.55 × 0.30 × 0.26 mm
*Z* = 4	

### Data collection

**Table:**

Rigaku Saturn diffractometer	2013 independent reflections
Radiation source: fine-focus sealed tube	1688 reflections with *I* > 2σ(*I*)
graphite	*R*_int_ = 0.032
Detector resolution: 14.63 pixels mm^-1^	θ_max_ = 27.5°, θ_min_ = 3.6°
ω scans	*h* = −18→21
Absorption correction: multi-scan (*REQAB*; Jacobson, 1998)	*k* = −9→16
*T*_min_ = 0.013, *T*_max_ = 0.030	*l* = −12→10
5341 measured reflections	

### Refinement

**Table:**

Refinement on *F*^2^	Secondary atom site location: difference Fourier map
Least-squares matrix: full	Hydrogen site location: inferred from neighbouring sites
*R*[*F*^2^ > 2σ(*F*^2^)] = 0.038	H-atom parameters constrained
*wR*(*F*^2^) = 0.080	*w* = 1/[σ^2^(*F*_o_^2^) + (0.0419*P*)^2^] where *P* = (*F*_o_^2^ + 2*F*_c_^2^)/3
*S* = 1.00	(Δ/σ)_max_ < 0.001
2013 reflections	Δρ_max_ = 1.56 e Å^−^^3^
107 parameters	Δρ_min_ = −2.20 e Å^−^^3^
0 restraints	Extinction correction: *SHELXL97* (Sheldrick, 2008), Fc^*^=kFc[1+0.001xFc^2^λ^3^/sin(2θ)]^-1/4^
Primary atom site location: structure-invariant direct methods	Extinction coefficient: 0.00186 (14)

### Special details

**Table:**

Geometry. All e.s.d.'s (except the e.s.d. in the dihedral angle between two l.s. planes) are estimated using the full covariance matrix. The cell e.s.d.'s are taken into account individually in the estimation of e.s.d.'s in distances, angles and torsion angles; correlations between e.s.d.'s in cell parameters are only used when they are defined by crystal symmetry. An approximate (isotropic) treatment of cell e.s.d.'s is used for estimating e.s.d.'s involving l.s. planes.
Refinement. Refinement of *F*^2^ against ALL reflections. The weighted *R*-factor *wR* and goodness of fit *S* are based on *F*^2^, conventional *R*-factors *R* are based on *F*, with *F* set to zero for negative *F*^2^. The threshold expression of *F*^2^ > σ(*F*^2^) is used only for calculating *R*-factors(gt) *etc*. and is not relevant to the choice of reflections for refinement. *R*-factors based on *F*^2^ are statistically about twice as large as those based on *F*, and *R*- factors based on ALL data will be even larger.

### Fractional atomic coordinates and isotropic or equivalent isotropic displacement parameters (Å^2^)

**Table:**

	*x*	*y*	*z*	*U*_iso_*/*U*_eq_	
Hg1	0.5000	0.24799 (3)	0.2500	0.03257 (15)	
Br1	0.37219 (4)	0.18078 (6)	0.00215 (7)	0.03794 (19)	
S1	0.63765 (10)	0.19500 (16)	0.09685 (16)	0.0354 (4)	
N1	0.5935 (3)	0.3774 (4)	0.1882 (5)	0.0300 (11)	
N2	0.5304 (3)	0.2847 (4)	−0.1745 (5)	0.0282 (11)	
C1	0.6470 (3)	0.3344 (5)	0.1311 (6)	0.0272 (13)	
C2	0.7060 (4)	0.3982 (7)	0.0995 (7)	0.0438 (18)	
H2A	0.7442	0.3669	0.0625	0.053*	
C3	0.7083 (4)	0.5050 (8)	0.1218 (7)	0.051 (2)	
H3	0.7469	0.5486	0.0984	0.062*	
C4	0.6537 (4)	0.5493 (6)	0.1790 (7)	0.0440 (16)	
H4	0.6530	0.6236	0.1937	0.053*	
C5	0.6000 (4)	0.4815 (6)	0.2142 (8)	0.0427 (16)	
H5	0.5657	0.5113	0.2598	0.051*	
C6	0.5593 (3)	0.1942 (5)	−0.1028 (6)	0.0284 (12)	
C7	0.5306 (4)	0.0926 (6)	−0.1734 (7)	0.0411 (15)	
H7	0.5525	0.0287	−0.1176	0.049*	

### Atomic displacement parameters (Å^2^)

**Table:**

	*U*^11^	*U*^22^	*U*^33^	*U*^12^	*U*^13^	*U*^23^
Hg1	0.0342 (2)	0.0283 (2)	0.0378 (2)	0.000	0.01890 (15)	0.000
Br1	0.0372 (3)	0.0317 (4)	0.0401 (3)	−0.0041 (3)	0.0137 (3)	0.0007 (3)
S1	0.0382 (7)	0.0330 (10)	0.0332 (7)	0.0155 (7)	0.0148 (6)	0.0078 (7)
N1	0.029 (2)	0.021 (3)	0.041 (2)	−0.003 (2)	0.018 (2)	0.000 (2)
N2	0.030 (2)	0.019 (3)	0.031 (2)	0.000 (2)	0.0111 (19)	−0.004 (2)
C1	0.018 (2)	0.035 (4)	0.025 (2)	0.003 (2)	0.007 (2)	0.008 (2)
C2	0.032 (3)	0.063 (6)	0.043 (3)	−0.013 (3)	0.023 (3)	−0.009 (3)
C3	0.050 (4)	0.059 (6)	0.045 (3)	−0.032 (4)	0.022 (3)	−0.004 (4)
C4	0.045 (3)	0.029 (4)	0.054 (4)	−0.012 (3)	0.019 (3)	−0.002 (3)
C5	0.041 (3)	0.028 (4)	0.066 (4)	−0.003 (3)	0.030 (3)	−0.004 (3)
C6	0.028 (3)	0.026 (4)	0.036 (3)	0.008 (2)	0.019 (2)	0.008 (3)
C7	0.066 (4)	0.015 (3)	0.048 (3)	0.005 (3)	0.030 (3)	0.010 (3)

### Geometric parameters (Å, °)

**Table:**

Hg1—N1	2.485 (5)	C2—C3	1.350 (12)
Hg1—N1^i^	2.485 (5)	C2—H2A	0.9400
Hg1—Br1	2.5056 (6)	C3—C4	1.371 (10)
Hg1—Br1^i^	2.5056 (6)	C3—H3	0.9400
S1—C1	1.767 (7)	C4—C5	1.373 (9)
S1—C6	1.773 (5)	C4—H4	0.9400
N1—C5	1.321 (9)	C5—H5	0.9400
N1—C1	1.349 (7)	C6—C7	1.417 (9)
N2—C6	1.299 (8)	C7—C7^ii^	1.365 (12)
N2—N2^ii^	1.347 (9)	C7—H7	0.9400
C1—C2	1.394 (9)		
			
N1—Hg1—N1^i^	98.8 (2)	C1—C2—H2A	120.0
N1—Hg1—Br1	108.55 (10)	C2—C3—C4	119.4 (6)
N1^i^—Hg1—Br1	96.77 (10)	C2—C3—H3	120.3
N1—Hg1—Br1^i^	96.77 (10)	C4—C3—H3	120.3
N1^i^—Hg1—Br1^i^	108.55 (10)	C3—C4—C5	117.8 (7)
Br1—Hg1—Br1^i^	140.84 (4)	C3—C4—H4	121.1
C1—S1—C6	99.7 (3)	C5—C4—H4	121.1
C5—N1—C1	117.4 (5)	N1—C5—C4	124.4 (6)
C5—N1—Hg1	126.8 (4)	N1—C5—H5	117.8
C1—N1—Hg1	115.6 (4)	C4—C5—H5	117.8
C6—N2—N2^ii^	119.5 (4)	N2—C6—C7	124.2 (5)
N1—C1—C2	120.9 (6)	N2—C6—S1	119.1 (5)
N1—C1—S1	117.0 (4)	C7—C6—S1	116.7 (5)
C2—C1—S1	122.0 (5)	C7^ii^—C7—C6	116.4 (3)
C3—C2—C1	120.0 (6)	C7^ii^—C7—H7	121.8
C3—C2—H2A	120.0	C6—C7—H7	121.8
			
N1^i^—Hg1—N1—C5	11.6 (4)	S1—C1—C2—C3	177.5 (5)
Br1—Hg1—N1—C5	111.9 (5)	C1—C2—C3—C4	1.5 (10)
Br1^i^—Hg1—N1—C5	−98.4 (5)	C2—C3—C4—C5	1.3 (10)
N1^i^—Hg1—N1—C1	−172.8 (4)	C1—N1—C5—C4	3.8 (9)
Br1—Hg1—N1—C1	−72.5 (3)	Hg1—N1—C5—C4	179.3 (5)
Br1^i^—Hg1—N1—C1	77.2 (3)	C3—C4—C5—N1	−4.1 (10)
C5—N1—C1—C2	−0.7 (8)	N2^ii^—N2—C6—C7	−0.2 (9)
Hg1—N1—C1—C2	−176.7 (4)	N2^ii^—N2—C6—S1	−178.8 (5)
C5—N1—C1—S1	180.0 (4)	C1—S1—C6—N2	1.6 (4)
Hg1—N1—C1—S1	3.9 (5)	C1—S1—C6—C7	−177.1 (4)
C6—S1—C1—N1	96.6 (4)	N2—C6—C7—C7^ii^	0.6 (10)
C6—S1—C1—C2	−82.8 (5)	S1—C6—C7—C7^ii^	179.2 (6)
N1—C1—C2—C3	−1.9 (9)		

Symmetry codes: (i) −*x*+1, *y*, −*z*+1/2; (ii) −*x*+1, *y*, −*z*−1/2.
